# Squamous cell carcinoma arising from chronic lymphedema: case report and review of the literature

**DOI:** 10.1590/S1516-31802010000100009

**Published:** 2010-01-07

**Authors:** Carlos Augusto Gomes, Camila Beatriz Silva Magalhães, Cleber Soares, Rodrigo de Oliveira Peixoto

**Affiliations:** I MD, PhD. Adjunct professor, Department of Surgery, Hospital Universitário (HU), Universidade Federal de Juiz de Fora (UFJF), and Monte Sinai Hospital, Juiz de Fora, Minas Gerais, Brazil.; II MD. General Surgery resident, Department of Surgery, Hospital Universitário (HU), Universidade Federal de Juiz de Fora (UFJF), Juiz de Fora, Minas Gerais, Brazil.; III MD. Attending physician, Department of Surgery, Hospital Universitário (HU), Universidade Federal de Juiz de Fora (UFJF), and Monte Sinai Hospital, Juiz de Fora, Minas Gerais, Brazil.; IV MD, MSc. Assistant professor, Department of Surgery, Hospital Universitário (HU), Universidade Federal de Juiz de Fora (UFJF), and Monte Sinai Hospital, Juiz de Fora, Minas Gerais, Brazil.

**Keywords:** Carcinoma, squamous cell, Lymphedema, Skin cancer, Angioedema, Neoplasms, glandular and epithelial, Carcinoma de células escamosas, Linfedema, Neoplasias cutâneas, Angioedema, Neoplasias epiteliais e glandulares

## Abstract

**CONTEXT:**

Squamous cell carcinoma arising from chronic lymphedema has only been reported in the literature 11 times (12 cases). Some aspects of its pathogenesis remain unclear and, for the first time, attention has been drawn to epidemiological data.

**CASE REPORT:**

A 90-year-old white female with chronic unilateral lower-limb lymphedema, secondary to trauma 20 years earlier, presented with a three-month history of a vegetating cutaneous lesion. There had not been any previous local ulceration. The tumor was completely excised and the histopathological analysis showed that it was an infiltrating squamous cell carcinoma. A literature review in the Medline (Medical Literature Analysis and Retrieval System Online) and Lilacs (Literatura Latino-Americana e do Caribe em Ciências da Saúde) databases using the MeSH (Medical Subject Heading) terms "Carcinoma, Squamous Cell" AND "Lymphedema" identified 112 references and found 12 similar case reports.

## RESUMO

**CONTEXTO:**

O crescimento de carcinoma de células escamosas a partir de linfedema crônico foi relatado apenas 11 vezes na literatura (12 casos). Aspectos sobre sua patogênese ainda não foram completamente elucidados e pela primeira vez chama-se atenção para os dados epidemiológicos.

**RELATO DE CASO:**

Paciente do sexo feminino, branca, 90 anos de idade, portadora de linfedema crônico unilateral de membro inferior secundário a trauma sofrido há 20 anos, apresentou história de lesão cutânea vegetante de três meses. Não havia relato de lesão local prévia. O tumor foi completamente excisado e sua análise histopatológica mostrou tratar-se de carcinoma escamocelular infiltrante. Uma revisão da literatura nos bancos de dados Medline (Medical Literature Analysis and Retrieval System Online) e Lilacs (Literatura Latino-Americana e do Caribe em Ciências da Saúde), utilizando-se dos termos MeSH (Medical Subject Heading) "Carcinoma, Squamous Cell" AND "Lymphedema", identificou 112 citações e encontrou relatos de 12 casos semelhantes.

## INTRODUCTION

Despite the known propensity of areas of congenital or acquired chronic lymphedema to develop malignancy, there are few reports in the literature correlating this with squamous cell carcinoma. Our review found only 11 such reports (12 cases, Table 1).^1-10^ There has been little discussion and emphasis on the epidemiological characteristics and carcinogenetic predisposition of chronic lymphadema.^1-3^ Tumor development in areas of chronic lymphedema is believed not to happen serendipitously,^1,2^ and inherited susceptibility to carcinogenic stimuli^4,5^ is thought to play a role. Knowledge about predisposing dermatological conditions and epidemiological features is very important, in order to diagnose the disease at an early stage.

**Table 1.** Epidemiological aspects of cases of squamous cell carcinoma arising from chronic lymphedema reported in the literature^2^

Reference	n	Years of age	Sex	Etiology	Location	Risk factor
1	2	52	Male	Trauma	Leg	Ultraviolet B therapy
		63	Female	Idiopathic	Leg	None
2	1	82	Female	Lymphadenectomy	Leg	None
3	1	69	Male	Trauma	Foot	None
4	1	53	Male	Congenital	Foot	Verrucous hyperplasia
5	1	88	Female	Post-mastectomy	Hand	Vitiligo
6	1	40	Male	Congenital	Leg	None
7	1	48	Female	Lymphedematous	Arm	Dystrophic bullous epidermolysis
8	1	79	Female	Post-mastectomy	Forearm	None
9	1	37	Male	Congenital	Foot	Verrucous hyperplasia
10	1	25	Female	Idiopathic	Subungual	Warts
Present report	1	90	Female	Trauma	Leg	None

## CASE REPORT

A 90-year-old white female with chronic unilateral lower-limb lymphedema, secondary to trauma 20 years earlier, presented with a three-month history of a vegetating cutaneous lesion measuring 1.5 × 1.2 cm, on the anteromedial face of her right leg. There had not been any previous local ulceration. Physical examination did not show any lymph node enlargement in the groin on the same side. Abdominal ultrasound and chest radiography did not show any organ involvement. The tumor was completely excised with one-centimeter surgical margins, and the wound was closed. Histopathological analysis on the biopsy specimen showed an infiltrating squamous cell carcinoma, with free surgical margins. A follow-up 20 months later showed controlled local disease.

## DISCUSSION

A review of the literature in the Medline (Medical Literature Analysis and Retrieval System Online) and Lilacs (Literatura Latino-Americana e do Caribe em Ciências da Saúde) databases using the MeSH (Medical Subject Heading) terms "Carcinoma, Squamous Cell" AND "Lymphedema" identified and found 11 similar case reports with 12 cases (Table 1).

Lymphedema may be primary or secondary to either disease or surgery. Primary lymphedema is sometimes congenital (Milroy’s disease), but it may also develop at any time of life, particularly during puberty. According to the literature search results, the etiology of squamous cell carcinoma associated with chronic lymphedema was congenital in three reported cases and secondary to acquired conditions in the others. In the review of the literature, acquired chronic lymphedema was classified according to its pathophysiological mechanism, as follows: post-lymphadenectomy in three instances, idiopathic in two, secondary to trauma in three and lymphedematous in one, as well as in the present case report. 67% of the cases were located in the lower limbs and 33% in the upper limbs (Table 1). In fact, secondary postsurgical lymphedema of the extremities is the most common form in developed countries.^4-6^

Eight patients (67%) were diagnosed during their active middle age. The time lapse from lymphedema to tumor diagnosis was not established in the case reports. In addition, the limited number of cases does not allow conclusions to be reached regarding the relationship between the duration of chronic lymphedema and the development of squamous cell carcinoma. A speculative but noteworthy trend towards late onset was seen among the patients with congenital etiology (37, 53 and 63 years). Conversely, the time taken for squamous cell carcinoma to develop in cases of acquired chronic lymphedema may range from a few years to decades. Slightly greater female incidence (59%) of squamous cell carcinoma and chronic lymphedema was noticed. Post-mastectomy lymphedema in two patients probably accounted for this difference.^2,5^

Although rarely found in areas of chronic lymphedema, squamous cell carcinoma is frequently related to chronic cicatricial areas, especially those due to burns (Marjolin’s ulcer). Among the additional dermatological risk factors (seen in 50% of the cases) for the development of squamous cell carcinoma are angiosarcoma, vulgar wart, chronic verrucous hyperplasia, wart-like epidermodysplasia, generalized vitiligo, dystrophic bullous epidermolysis and ultraviolet B (UVB) therapy (Table 1). Like in other cases reported in the literature,^1-3,6,8^ no other additional factor was noticed in our case. Some theories attempting to explain a putative mechanism have been put forward. Futrell and Myers highlighted the immunological status governing the response of animal hosts to skin-implanted tumors, with or without an intact lymphatic system. It was noticed, in their study, that although the tumoral solution failed to induce malignancy when injected into an area where the lymphatic vessels had been spared, large and lethal tumors developed whenever the injection was in an area where the lymphatics had been impaired.^11^

It is also believed that deficiencies in the lymphatic drainage system hamper early recognition of tumor-specific antigens.^1^ Chronic stasis, thereby producing local changes in the lymphatic protein composition (decreased alpha-2 globulin fraction and increased albumin-globulin ratio) and delaying protein transportation from the interstitial space into the lymphatic tissue, might change the tissue antigenic composition and/or regional immunological competence.^4-6^

The treatment of choice is complete surgical resection of the tumor with free margins, associated with ipsilateral lymphadenectomy in cases of lymph node metastasis. Radiation therapy and chemotherapy are used as adjunctive approaches.^4,6,12^ This calls for periodic follow-up of predisposed subjects, with the aim of early diagnosis and adequate surgical management.

Every patient suffering from chronic lymphedema needs to be educated about the importance of good hygiene, regular follow-up, and elevation and elastic compression of the affected limb.
